# Consumption of Korean Foods with High Flavonoid Contents Reduces the Likelihood of Having Elevated C-Reactive Protein Levels: Data from the 2015–2017 Korea National Health and Nutrition Examination Survey

**DOI:** 10.3390/nu11102370

**Published:** 2019-10-04

**Authors:** Dongwoo Ham, Shinyoung Jun, Minji Kang, Hee-Young Paik, Hyojee Joung, Sangah Shin

**Affiliations:** 1 Institute of Health and Environment, Seoul National University, Seoul 08826, Korea; dwhampch@snu.ac.kr; 2 Department of Nutrition Science, Purdue University, West Lafayette, IN 47907, USA; jun24@purdue.edu; 3 Center for Gendered Innovations in Science and Technology Research (GISTeR), Korea Federation of Women’s Science & Technology Associations, Seoul 06130, Korea; mkang@cc.hawaii.edu (M.K.); hypaik@kofwst.org (H.-Y.P.); 4 Cancer Epidemiology Program, University of Hawaii Cancer Center, Honolulu, HI 96813, USA; 5 Department of Public Health, Graduate School of Public Health, Seoul National University, Seoul 08826, Korea; 6 Department of Food and Nutrition, College of Biotechnology and Natural Resources, Chung-Ang University, Gyeonggi-do 17546, Korea

**Keywords:** C-reactive protein, flavonoids, Korea National Health and Nutrition Examination Survey, Korean food, total antioxidant capacity

## Abstract

This study was conducted to investigate associations between C-reactive protein (CRP) levels and Korean food (KF) consumption and flavonoid intake from the 2015–2017 Korea National Health and Nutrition Examination Survey. A total of 6025 men and 8184 women (≥19 years) who completed a 24-h dietary recall and health examination were analyzed. The individual KF consumption rate was defined as the proportion of KF of total food consumed and categorized into tertiles. Odds ratios (ORs) for elevated CRP levels (>3.0 mg/L) according to KF consumption rate and flavonoid intake/dietary total antioxidant capacity (TAC) (<median; ≥median) were obtained by multiple logistic regression. KF consumption was inversely associated with CRP levels in women (*p* = 0.0236) and positively associated with flavonoid intake/dietary TAC in both sexes (*p* < 0.0001). Compared to women who consumed less than the median amount of flavonoid or TAC with KF consumption rates in the lowest tertile, those who consumed more flavonoid (OR = 0.59, 95% CI 0.42–0.83) or TAC (OR = 0.58, 95% CI 0.41–0.82) in the highest tertile showed significantly lower ORs for elevated CRP levels. Thus, consuming KFs rich in flavonoid is effective for regulating CRP levels.

## 1. Introduction

Inflammation is an early step in immunity to protect against metabolic disturbances caused by infection [1]. Immune cells involved in inflammatory reaction destroy antigens by producing reactive oxygen species (ROS), but excessive accumulation of ROS due to prolonged inflammation can cause oxidative stress [2,3]. Oxidative stress can injure tissues, affect intracellular metabolism of proteins or lipids, and induce mutations in mitochondrial DNA, which can lead to non-communicable diseases (NCDs) such as obesity and metabolic syndrome [4].

As a consequence of inflammation, inflammatory markers may be expressed [5]. C-reactive protein (CRP) is a predominant inflammatory marker produced in the liver that can itself contribute to the expression of other inflammatory markers such as tumor necrosis factor-α and interleukin-6 [2,6,7]. CRP levels have been positively associated with increased oxidative stress [8] and cardiovascular diseases such as atherosclerosis and hypertension [2,7,9]. As the prevalence of hypertension and dyslipidemia continues to rise [10] and cardiovascular diseases have come to be the second leading cause of deaths for Koreans in 2017 [11], it is necessary to elucidate the various factors that affect expression and regulation of CRP.

Oxidative stress can be regulated by antioxidants, which react with ROS [12,13,14,15,16,17]. Antioxidants are usually obtained from foods, and major dietary antioxidants include vitamins (A, C, E) and phytochemicals such as flavonoids [18]. Dietary antioxidants are present primarily in plant-derived foods, including vegetables and fruits [19]. According to previous studies conducted in the US, CRP levels were inversely associated with flavonoid intake, as well as vegetables and fruits consumption [20,21]. In Korea, some cross-sectional studies have shown a positive relationship between a dietary inflammatory index and CRP levels [22,23], but associations of CRP levels with individual flavonoids based on nationally representative data have not been determined.

The merits of a traditional Korean diet have been examined recently [24], as the dietary patterns of Koreans have been rapidly westernized to include a high level of fat, and NCDs have become a major public health issue [25]. According to an intervention study, consumption of Korean foods (KFs) effectively reduced heart rates and concentrations of glycated hemoglobin in Korean patients with hypertension or diabetes [26]. Another intervention study conducted in the US reported improvements in low-density lipoprotein cholesterol and total cholesterol levels after KF consumption among American adults [27].

Such beneficial effects of consuming KFs can be attributed to the high content of plant-derived ingredients. According to analyses of the 2007–2012 Korea National Health Examination and Nutrition Survey (KNHANES), the most frequently consumed KFs were vegetables, grains, legumes, and fruits [24]. Using the same data, another study showed that consumption of fruits, vegetables, and legumes contributed the most to the flavonoid intake and dietary total antioxidant capacity (TAC) of Koreans [28,29]. Based on these results, it can be assumed that KFs rich in plant-derived ingredients are highly correlated with the level of antioxidant phytochemical intake. Thus, we hypothesized that KF consumption reduces oxidative stress and regulates the expression of inflammatory markers.

Several studies have been conducted to investigate the health benefits of KF consumption and flavonoid intake. However, to the best of our knowledge, associations between blood CRP levels and both KF consumption and flavonoid intake have not been determined. Therefore, we aimed to explore associations between CRP levels and KF consumption and flavonoid intake by analyzing data from the 2015–2017 KNHANES.

## 2. Materials and Methods

### 2.1. Study Design and Population

The KNHANES is a national cross-sectional survey that is conducted every year by the Korea Centers for Disease Control and Prevention. The KNHANES applies a complex sampling design with strata, clusters, and weights to represent the Korean population and collects data on demographic variables, health-related behaviors, health status, and dietary intake using a 24-h recall and food frequency questionnaire. Detailed information about the KNHANES is available elsewhere [30].

Among the 23,657 subjects who participated in the 2015–2017 KNHANES, we excluded those who were younger than 19 years (*n* = 4812), whose CRP levels were missing or outside of the detection limits (<0.15 mg/L or >20.0 mg/L) (*n* = 2205) [31], or who reported extremely low or high daily energy intake (<500 kcal/day or ≥5000 kcal/day) (n = 2431) [32]. Finally, a total of 6025 men and 8184 women were included in the analyses.

Approval from institutional review board of the 2015–2017 KNHANES was waived because this study was conducted for public welfare by the Korean government according to the Bioethics and Safety Act and the Enforcement Rule within that Act. All subjects provided written informed consent before participation.

### 2.2. Estimation of KF Consumption Rate

The KF consumption rate was estimated using a KF database that was constructed in a previous study [33]. The KF database is based on results of a survey conducted in 2011–2012 to identify perceptions of 1322 food items commonly consumed in Korea by 117 experts in the fields of food science, nutrition, or cooking and 562 adults residing in the capital area (Seoul and Gyeonggi Province) [34,35]. In 2014, a focus group of 14 experts assembled a systematic database by reviewing and thoroughly discussing those survey results to reach a final decision about whether each food should be categorized as a KF or not. The focus group defined KF as follows: (1) if the main ingredient or recipe originated from Korea; (2) if the main ingredient of a food was used in Korea for a long time, even if the ingredient was not indigenous; (3) if a food was consumed only in Korea, even if its main ingredient was not indigenous; and (4) if a food was developed or widely consumed in Korea, even if the recipe was not traditional [33].

The KF database was linked to the 24-h dietary recall data of subjects in our study. The KF consumption rate was calculated as the proportion of KF among all food consumed by a subject in a single day.

### 2.3. Assessment of Individual Flavonoid Intake and Dietary TAC

Individual flavonoid intake was estimated by linking information in the flavonoid composition database to food consumption data of the subjects. The flavonoid composition database included individual amounts of flavonols (isorhamnetin, kaempferol, myricetin, quercetin), flavones (apigenin, luteolin), flavanones (eriodictyol, hesperetin, naringenin), flavan-3-ols (catechin, epicatechin, epigallocatechin, theaflavin, theaflavin-3-gallate, theaflavin 3′-gallate, theaflavin 3,3′-digallate, thearubigin), isoflavones (daidzein, genistein, glycetin), anthocyanidins (cyanidin, delphinidin, malvidin, pelargonidin, peonidin, petunidin), and proanthocyanidins (dimers, trimers, 4–6 monomers, 7–10 monomers, 10+ monomers) in 3193 foods commonly consumed in Korea. Each value was derived from the databases of the Korea Rural Development Administration [36], United States Department of Agriculture (USDA) [37,38,39], or France National Institute of Agricultural Research (INRA) [40,41,42]. The flavonoid composition database covered 63.4% of food items and 60.3% of food consumption of the subjects. Some examples of KFs with high flavonoid contents include fast-fermented bean paste (‘*cheonggukjang*’, 167.79 mg/100 g), pickled radish (157.52 mg/100 g), water parsley (147.81 mg/100 g), and dried persimmon (120.19 mg/100 g). Detailed information about the construction of the flavonoid composition database is available elsewhere [29].

In addition, we used the TAC database to assess the daily dietary TAC of the subjects. The TAC values (vitamin C equivalent, VCE) for each food in the TAC database were calculated according to the method introduced by Floegel et al. in 2010 [43] using the flavonoid composition database [29] and the antioxidant vitamin composition database [44,45]. Individual dietary TACs represented the sum of TACs in whole foods consumed by each subject. The TAC database covered 87.4% of food items and 91.8% of food consumption of the subjects. Construction of the TAC database is described in detail in a previous report [28].

### 2.4. Analysis of Elevated CRP Levels and Determination of Obese Subjects

The analysis of blood CRP levels and anthropometric measurements were conducted during health examinations by well-trained technicians after subjects had fasted for at least 8 h. The analysis of blood CRP levels was based on immunoturbidimetry measures collected using Cobas (Roche, Germany), and elevated CRP levels were defined as those >3.0 mg/L [5]. Body mass index (BMI) was obtained from height (m) and weight (kg), and subjects with BMIs ≥25 kg/m^2^ were defined as obese [46]. Information about the laboratory analyses and anthropometric measurements is available elsewhere [31].

### 2.5. Demographic Variables and Health-Related Behaviors

Each subject was asked to complete an interview and a self-reported questionnaire regarding demographic variables (age, household income, education level) and health-related behaviors (current smoking, regular alcohol consumption, physical activity). Household income was categorized into quartiles of monthly household income (low, middle-low, middle-high, high). Education level was classified as elementary school or less, middle school, high school, and college or more. Current smoking was considered “yes” for those who had smoked ≥100 cigarettes over a lifetime and were still smoking. Regular alcohol consumption was considered “yes” if the subject had drunk alcohol more than once per month over the past year. Physical activity was considered “yes” for those who had performed vigorous-intensity activities for ≥1.25 h/week or intermediate-intensity activities for ≥2.5 h/week.

### 2.6. Statistical Analyses

All analyses were stratified by the sex of the subjects. Based on the complex sampling design of the KNHANES, we applied strata, clusters, and weights to the statistical models. All analyses were conducted using Statistical Analysis System software version 9.4 (SAS 9.4, SAS Institute, Cary, NC, USA). A two-sided *p*-value <0.05 was considered statistically significant.

General characteristics of the subjects according to CRP statuses (normal or elevated) and tertile groups of KF consumption rates were presented as percentages for categorical variables and means ± standard errors for continuous variables. CRP levels and BMIs of each group were estimated by least-square means adjusted for age (continuous), household income, education level, current smoking, regular alcohol consumption, and physical activity as confounding variables. Rao–Scott chi-square tests and generalized linear model (GLM) analyses were used to test the statistical significance of differences between groups for categorical and continuous variables, respectively. The daily flavonoid intake and dietary TAC of each subject were estimated per 1000 kcal to minimize the effects of energy consumption. We compared flavonoid intakes and dietary TACs of each KF consumption rate tertile group. *P*-values for linear trends of flavonoid intake and dietary TAC by tertile group were estimated based on the median values of KF consumption rates for each group. The subjects were further categorized into two levels based on flavonoid intake and dietary TAC (<median, ≥median). Considering the KF consumption rate tertile groups and flavonoid intake levels simultaneously, odds ratios (ORs) and 95% confidence intervals (CIs) for elevated CRP levels in each group were obtained by multiple logistic regression analysis, with adjustments for age (continuous), household income, education level, current smoking, regular alcohol consumption, and physical activity. The subjects with KF consumption rates in the lowest tertile and flavonoid intakes less than the median were treated as the reference group.

## 3. Results

### 3.1. General Characteristics of Subjects According to CRP Status and KF Consumption Rate Tertile Group

General characteristics of the subjects according to CRP status are presented in Table 1. The prevalence of elevated CRP levels was 9.5% in men (572/6025) and 7.2% in women (588/8184), respectively. For both men and women, subjects with elevated CRP levels tended to perform less physical activity (*p* = 0.0030 for men; *p* = 0.0040 for women) and be more obese than those with normal CRP levels (*p* < 0.0001 for both sexes). Among men, elevated CRP levels were associated with lower household incomes (*p* < 0.0001) and lower education levels (*p* = 0.0002) relative to normal CRP levels, but the proportion of subjects reporting regular alcohol consumption was higher among those with normal CRP levels than those with elevated CRP levels (*p* = 0.0363).

In Table 2, general characteristics according to KF consumption rate tertile group for each sex are presented. Subjects with higher KF consumption rates tended to be older (*p* < 0.0001 for both sexes), have lower household incomes (*p* < 0.0001 for both sexes), and lower education levels (*p* < 0.0001 for both sexes), smoke less currently (*p* = 0.0013 for men; *p* = 0.0295 for women), and perform less physical activity (*p* = 0.0048 for men; *p* < 0.0001 for women). Among women, the proportions of regular alcohol consumption decreased as KF consumption rate increased (*p* < 0.0001), but men showed no significant association. CRP levels had an inverse relationship with KF consumption rate in women (*p* for trend = 0.0236) but not in men.

### 3.2. Flavonoid Intake and Dietary TAC According to KF Consumption Rate Tertile Group

Table 3 shows dietary flavonoid intake and TAC, as well as macronutrient intake, according to KF consumption rate tertile group. Daily energy intake and amount of energy from fats decreased significantly as the KF consumption rate increased, whereas the amount of energy from carbohydrates increased with KF consumption rate in both sexes (*p* for trend < 0.0001). For both men and women, total flavonoid intake and dietary TAC were higher when they had higher KF consumption rates (*p* for trend < 0.0001). Among the subclasses of flavonoids, flavonol, isoflavone, and proanthocyanidin intake was positively associated with the KF consumption rate in both men and women (*p* for trend < 0.0001). Anthocyanidin intake, however, showed a positive relationship with KF consumption rate only in women (*p* for trend < 0.0001). In addition, both men and women with higher KF consumption rates consumed less flavanones than those with lower KF consumption rates (*p* for trend = 0.0028 for both sexes).

### 3.3. Associations between Elevated CRP Levels and KF Consumption Rate and Flavonoid Intake

**Figure 1:**
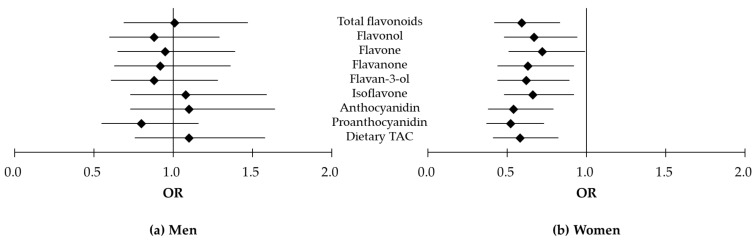
Odds ratios (ORs) for elevated C-reactive protein levels in subjects whose consumption of flavonoid or dietary total antioxidant capacity (TAC) was greater than the median value and who were in the highest tertile of Korean food (KF) consumption rate compared to those with lower flavonoid intake or dietary TAC who were in the lowest tertile of KF consumption rate: (**a**) Men; (**b**) Women.

Multivariable-adjusted ORs and 95% CIs for elevated CRP levels according to KF consumption rate and flavonoid intake (<median, ≥median) are indicated in Table 4 and Figure 1. Women in the highest tertile (T3) of KF consumption rate and who consumed total flavonoids or dietary TAC more than the median value had significantly lower ORs for elevated CRP levels than those in the lowest tertile (T1) of KF consumption rate and who consumed total flavonoids or dietary TAC less than the median value (OR = 0.59, 95% CI 0.42–0.83 for total flavonoids; OR = 0.58, 95% CI 0.41–0.82 for dietary TAC). Likewise, inverse associations between elevated CRP levels and KF consumption rate and flavonoid intake were observed for each subclass of flavonoids: OR = 0.67, 95% CI 0.48–0.94 for flavonol; OR = 0.72, 95% CI 0.51–0.99 for flavone; OR = 0.63, 95% CI 0.44–0.92 for flavanone; OR = 0.62, 95% CI 0.44–0.89 for flavan-3-ol; OR = 0.66, 95% CI 0.48–0.92 for isoflavone; OR = 0.54, 95% CI 0.38–0.79 for anthocyanidin; and OR = 0.52, 95% CI 0.37–0.73 for proanthocyanidin. Moreover, in the case of women who consumed less than the median value of flavanones, ORs for elevated CRP levels decreased as KF consumption rates increased (T2 vs. T1, OR = 0.71, 95% CI 0.51–0.99; T3 vs. T1, OR = 0.67, 95% CI 0.48–0.96). Meanwhile, men whose anthocyanidin intake was less than the median value for the highest tertile KF consumption rate had higher ORs for elevated CRP levels (OR = 1.52, 95% CI 1.04–2.22).

## 4. Discussion

In this study, we investigated associations between blood CRP levels and KF consumption and flavonoid intake by Korean adults using nationally representative data. For both sexes, subjects with higher KF consumption rates had significantly higher flavonoid intake and dietary TAC than those with lower KF consumption rates. Among women but not men, those with higher KF consumption rates had lower CRP levels. Furthermore, ORs for elevated CRP levels in women were significantly lower when their KF consumption and flavonoid intake were higher.

KF consumption rates were positively associated with total flavonoid intake for both sexes in this study. This result is consistent with the assumption that KFs are rich in flavonoids [24,29]. In particular, intake of flavonol, isoflavone, and proanthocyanidin increased in proportion to the KF consumption rate in both men and women. According to Jun et al. [29], Korean adults consumed flavonol mainly from vegetables, isoflavone from legumes and legume products, and proanthocyanidin from fruits and grains. They further analyzed the food items that contributed the most to flavonoid intake. Flavonol was mainly ingested from onions, radish leaves, and radishes. Tofu and soybeans were the major sources of isoflavone, and proanthocyanidin intake was mostly related to consumption of apples, grapes, and sorghum. In addition, Kim et al. [24] reported that Korean adults with higher KF consumption rates consumed legumes and legume products, vegetables, and fruits significantly more than those with lower KF consumption rates. Moreover, multigrain rice, fermented soybean paste stew (‘*doenjang jjigae*’), young radish kimchi, sliced radish kimchi (‘*kkakdugi*’), apples, and soybean sprouts, which were all reported to be rich in flavonoids, ranked among the top 20 most frequently consumed KFs.

In the current study, dietary TAC as well as total flavonoid intake increased when the KF consumption rates of both men and women increased. Dietary TAC is a comprehensive measure of antioxidant consumption that incorporates the different amounts of oxidative stress regulated by each antioxidant [28]. Various antioxidants obtained from foods have been investigated for their interactions with one another and cumulative or synergistic effects [19,43]. In a previous study on antioxidant vitamin intake according to KF consumption rate [24], nutrient densities of vitamin A (both retinol equivalents and retinol activity equivalents) and vitamin C were significantly higher among subjects in the highest KF consumption rate quartile than among those in the lowest quartile. Because KF consumption showed positive associations with both flavonoids and antioxidant vitamin intake, the contribution of KF consumption to dietary TAC has important implications for public health. Jun and her colleagues [28] found that major sources of TAC for Koreans were fruits, vegetables, and legumes and legume products, and that most of the top 20 foods that were major contributors to dietary TAC in Korean adults were frequently consumed KFs such as tangerines, apples, radishes, soybeans, and cabbage kimchi [24,28]. Based on the positive associations of KF consumption with flavonoid intake and dietary TAC observed in the present study, we further analyzed the contribution ratios to flavonoid intake and dietary TAC of the KFs consumed by the subjects and found that fruits (apple, grape, strawberry, persimmon, and peach), multigrain rice, soybean paste stew, cabbage kimchi, and green tea were the major contributing KFs to flavonoid intake and dietary TAC. Thus, our finding that higher KF consumption rates correlate with higher flavonoid intake and dietary TAC is consistent with the results of previous studies.

In this study, flavanone intake was significantly lower in the highest tertile KF consumption rate than in the lowest tertile for both sexes, unlike other flavonoids and dietary TAC. According to Jun et al. [29], 99.7% of the flavanone ingested by Koreans is from fruits, with oranges (6.5%), fruit drinks (1.8%), and grapefruits (0.6%), which were not regarded as KFs [33]. Tangerines, which accounted for 90.3% of the flavanone intake by Koreans, were categorized as a KF. However, the effects of tangerine consumption on flavanone intake in our study might not be significant because of seasonal variation in tangerine consumption; generally, consumption is limited to the winter season in Korea [47].

Among women, an inverse association between elevated CRP levels and KF consumption and flavonoids intake was found. Previous studies have reported inverse relationships between flavonoid intake or dietary TAC and CRP levels. According to a study of premenopausal American women, higher isoflavone intake correlated with lower CRP levels [48]. In a study of young Japanese women, dietary TAC, which was mainly attributable to tea consumption, was inversely associated with CRP levels [49]. Moreover, consumption of strawberries and cranberry juice, both with high levels of flavonoids, showed an association with low CRP levels in Americans, based on a prospective study [50] and randomized controlled trial [51], respectively. However, no pervious study evaluated CRP levels among Koreans according to KF consumption rate and flavonoid intake. Thus, this study provides a basis for future studies on the beneficial effects of KFs with high flavonoid contents and dietary TAC on the regulation of oxidative stress and inflammatory biomarkers.

In the case of flavanones, ORs for elevated CRP levels among women in the second and third tertiles of KF consumption were significantly lower, even though their flavanone intake was less than the median value. This finding might reflect the inverse relationship between KF consumption and flavanone intake in the present study. Moreover, effects of flavanone intake on health status have been reported previously, but the results of these studies are inconsistent. According to a prospective study conducted by Goetz et al. [52], the hazard ratio from ischemic stroke among American adults showed significantly negative relationships with flavanone intake and consumption of citrus or citrus juice. Rohrmann et al. [53] analyzed data from the Multiethnic Cohort to determine the relationship between flavonoid intake and inflammatory biomarkers and reported that consumption of flavanone could increase adiponectin and IL-6. A recent randomized crossover trial conducted in the UK showed that consumption of orange juice increased plasma flavanone levels, but no metabolic indices or levels of biomarker related to cardiovascular diseases were significantly affected [54]. Additional prospective studies are required to elucidate the associations between flavanone intake and expression of inflammatory markers.

In this study, positive relationships between KF consumption and flavonoid intake were observed for both sexes, but CRP levels were negatively associated with KF consumption only in women. Similarly, ORs for elevated CRP levels had significant inverse relationships with KF consumption rates and flavonoid intake only among women. Sex differences in relationships between KF consumption and CRP levels could be attributed to differences in flavonoid intake by each sex. In our study, mean nutrient densities of total flavonoids, as well as flavone, flavanone, flavan-3-ol, anthocyanidin, and proanthocyanidin, and dietary TAC in men in the highest tertile KF consumption rate were found to be lower than those in the lowest tertile in women. Lower flavonoid intake and dietary TAC in men compared to women might be because Korean men tend to consume less vegetables and fruits than women. Lee et al. [55] reported that the proportion (4.4%) of Korean men who fulfilled recommendations of consumption of both fruits and vegetables was significantly lower than that of women (6.1%) who achieved this goal, based on analyses of KNHANES data. According to analyses of 209,598 Korean adults in a nationwide cohort [56], more women (23.7%) than men (14.5%) answered that they consumed meals with mainly vegetables.

This study had some limitations. First, 24-h dietary recall might not represent the actual dietary behaviors of the subjects because of within-person variation over time. To reflect usual intake, food consumption data must be collected over a longer period of time. Second, causal associations between KF consumption and flavonoid intake and blood CRP levels could not be investigated because of the cross-sectional design. Therefore, additional studies with a prospective design are needed to elucidate the effects of KF consumption and flavonoid intake on CRP levels. Third, flavonoid intake and dietary TAC of the subjects could be underestimated because flavonoid composition and TAC databases did not account for all foods consumed. Fourth, only blood CRP levels were measured as an inflammatory biomarker; other biomarkers exist that might have more significant associations with dietary antioxidant intake. Thus, various biomarkers of inflammation or oxidative stress should be evaluated in future studies. Finally, other beneficial effects of KF consumption, such as low-calorie or low-fat contributions to the diet of a person, were not considered in this study. However, we considered the nutrient density per 1000 kcal as an independent variable to adjust for potential confounding effects of energy intake. In future studies, a more comprehensive approach that takes into account various aspects of KF consumption is needed. Despite these limitations, to the best of our knowledge, this study is the first to determine associations between CRP levels and KF consumption and flavonoid intakes using nationally representative survey data.

## 5. Conclusions

In conclusion, KF consumption was positively associated with flavonoid intake and dietary TAC in Korean adults. Blood CRP levels showed an inverse relationship with KF consumption rate only among women. These findings indicate that consumption of KFs mainly composed of fruits, grains, and legume products with high flavonoid contents and high dietary TAC might be effective in regulating oxidative stress and the expression of inflammatory markers in humans. Additional studies with a prospective design are required to elucidate the causal relationship between KF consumption and inflammatory marker levels and to establish strategies to promote the positive aspects of KFs globally.

## Figures and Tables

**Table 1:** General characteristics of subjects according to C-reactive protein (CRP) status ^1^.

Variables	Men		*p*-Value ^3^	Women		*p*-Value ^3^
	Normal CRP	Elevated CRP ^2^		Normal CRP	Elevated CRP ^2^	
N	5453	572		7596	588	
C-reactive protein (mg/L), mean ± SE ^4^	0.79 ± 0.01	6.16 ± 0.18	<0.0001	0.70 ± 0.01	5.75 ± 0.13	<0.0001
Korean food consumption rate (%), mean ± SE	64.90 ± 0.32	66.56 ± 0.93	0.0790	64.65 ± 0.30	63.75 ± 0.84	0.2977
Age (years), n (%)						
19–29	676 (12.4)	46 (8.0)	<0.0001	787 (10.4)	65 (11.1)	0.0433
30–49	1738 (31.9)	140 (24.5)		2698 (35.5)	184 (31.3)	
50–64	1565 (28.7)	169 (29.6)		2228 (29.3)	153 (26.0)	
≥65	1474 (27.0)	217 (37.9)		1883 (24.8)	186 (31.6)	
Household income, n (%) ^5^						
Low	932 (17.2)	163 (28.6)	<0.0001	1499 (19.8)	149 (25.4)	0.0518
Middle-low	1337 (24.6)	150 (26.3)		1872 (24.7)	151 (25.8)	
Middle-high	1507 (27.8)	117 (20.5)		2062 (27.2)	153 (26.1)	
High	1651 (30.4)	140 (24.6)		2139 (28.3)	133 (22.7)	
Education level, n (%)						
≤Elementary school	827 (16.1)	129 (23.8)	0.0002	1851 (25.7)	186 (33.4)	0.0664
Middle school	540 (10.5)	75 (13.9)		741 (10.3)	50 (9.0)	
High school	1739 (33.9)	166 (30.7)		2197 (30.4)	162 (29.1)	
≥College	2025 (39.5)	171 (31.6)		2428 (33.6)	159 (28.6)	
Current smoking, yes, n (%) ^6^	1771 (33.0)	194 (34.8)	0.1287	322 (4.3)	36 (6.2)	0.1621
Regular alcohol consumption, yes, n (%) ^7^	3802 (70.8)	372 (66.4)	0.0363	3067 (41.0)	214 (37.0)	0.0610
Physical activity, yes, n (%) ^8^	2469 (48.2)	226 (42.0)	0.0030	3087 (42.8)	205 (36.8)	0.0040
Obesity, yes, n (%) ^9^	2158 (39.6)	276 (48.3)	<0.0001	2261 (29.8)	302 (51.4)	<0.0001
Body mass index (kg/m^2^), mean ± SE ^4^	24.47 ± 0.06	25.81 ± 0.22	<0.0001	23.17 ± 0.05	25.79 ± 0.26	<0.0001

^1^ All analyses accounted for the complex sampling design effect and considered strata, clusters, and weights. ^2^ Elevated CRP levels were defined as those >3.0 mg/L. ^3^ *P*-values were obtained from Rao–Scott chi-square tests for categorical variables and generalized linear model (GLM) analyses for continuous variables. ^4^ Data are presented in least square means ± standard error adjusted for age (continuous), household income, education level, current smoking, regular alcohol consumption, and physical activity. ^5^ Household income was categorized according to quartiles: low (first quartile), middle-low (second quartile), middle-high (third quartile), and high (fourth quartile). ^6^ Current smoking was defined as having smoked ≥100 cigarettes over a lifetime and still smoking. ^7^ Regular alcohol consumption was defined as having drunk alcohol more than once a month over the past year. ^8^ Physical activity was defined as having performed vigorous-intensity activities for ≥1.25 h/week or intermediate-intensity activities for ≥2.5 h/week. ^9^ Obesity was defined as a body mass index ≥25 kg/m^2^.

**Table 2:** General characteristics of subjects according to Korean food (KF) consumption rate tertile groups ^1^.

Variables	Men			*p*-Value ^3^	Women			*p*-Value ^3^
	KF Consumption Rate Tertile ^2^				KF Consumption Rate Tertile ^2^			
	T1	T2	T3		T1	T2	T3	
N	1976	2040	2009		2730	2909	2545	
KF consumption rate (%), mean (range)	48.0 (0.0, 61.9)	69.3 (62.5, 76.7)	85.1 (76.9, 100.0)		46.9 (0.0, 60.9)	68.1 (61.1, 75.0)	84.9 (75.8, 100.0)	
Age (years), n (%)								
19–29	450 (22.8)	171 (8.4)	101 (5.0)	<0.0001	535 (19.6)	228 (7.8)	89 (3.5)	<0.0001
30–49	844 (42.7)	624 (30.6)	410 (20.4)		1275 (46.7)	1044 (35.9)	563 (22.1)	
50–64	378 (19.1)	651 (31.9)	705 (35.1)		559 (20.5)	918 (31.6)	904 (35.5)	
≥65	304 (15.4)	594 (29.1)	793 (39.5)		361 (13.2)	719 (24.7)	989 (38.9)	
Household income, n (%) ^4^								
Low	270 (13.7)	356 (17.5)	469 (23.5)	<0.0001	391 (14.4)	564 (19.5)	693 (27.3)	<0.0001
Middle-low	473 (24.1)	473 (23.2)	541 (27.1)		648 (23.8)	717 (24.7)	658 (25.9)	
Middle-high	584 (29.7)	565 (27.8)	475 (23.8)		779 (28.6)	801 (27.6)	635 (25.0)	
High	640 (32.5)	641 (31.5)	510 (25.6)		905 (33.2)	816 (28.2)	551 (21.7)	
Education level, n (%)								
≤Elementary school	163 (8.6)	332 (17.2)	461 (24.8)	<0.0001	366 (14.0)	683 (24.8)	988 (41.1)	<0.0001
Middle school	111 (5.9)	240 (12.5)	264 (14.2)		151 (5.8)	318 (11.6)	322 (13.4)	
High school	707 (37.5)	623 (32.3)	575 (30.9)		853 (32.5)	867 (31.5)	639 (26.6)	
≥College	905 (48.0)	732 (38.0)	559 (30.1)		1251 (47.7)	882 (32.1)	454 (18.9)	
Current smoking, yes, n (%) ^5^	706 (36.2)	696 (34.7)	563 (28.6)	0.0013	144 (5.4)	128 (4.5)	86 (3.5)	0.0295
Regular alcohol consumption, yes, n (%) ^6^	1399 (71.7)	1387 (69.1)	1388 (70.4)	0.5179	1280 (47.5)	1191 (41.5)	810 (32.5)	<0.0001
Physical activity, yes, n (%) ^7^	958 (51.0)	900 (46.7)	837 (45.2)	0.0048	1200 (45.8)	1168 (42.5)	924 (38.6)	<0.0001
Obesity, yes, n (%) ^8^	815 (41.3)	837 (41.1)	782 (39.0)	0.8242	763 (28.0)	895 (30.8)	905 (35.6)	<0.0001
				*p* for Trend ^9^				*p* for Trend ^9^
Body mass index (kg/m^2^), mean ± SE ^10^	24.48 ± 0.09	24.69 ± 0.10	24.61 ± 0.10	0.2835	23.33 ± 0.08	23.34 ± 0.08	23.41 ± 0.10	0.5343
C-reactive protein (mg/L), mean ± SE ^10^	1.21 ± 0.05	1.32 ± 0.06	1.26 ± 0.06	0.4364	1.10 ± 0.04	1.05 ± 0.03	0.98 ± 0.03	0.0236

^1^ All analyses accounted for the complex sampling design effect and considered strata, clusters, and weights. ^2^ The KF consumption rate was calculated as the proportion of KF among all food consumed by a subject in a single day and stratified into tertile groups. ^3^ *p*-values were obtained from Rao–Scott chi-square tests. ^4^ Household income was categorized according to quartiles: low (first quartile), middle-low (second quartile), middle-high (third quartile), and high (fourth quartile). ^5^ Current smoking was defined as having smoked ≥100 cigarettes over a lifetime and still smoking. ^6^ Regular alcohol consumption was defined as having drunk alcohol more than once a month over the past year. ^7^ Physical activity was defined as having performed vigorous-intensity activities for ≥1.25 h/week or intermediate-intensity activities for ≥2.5 h/week. ^8^ Obesity was defined as a body mass index ≥25 kg/m^2^. ^9^ *P* for trend values were obtained based on the median value for the KF consumption rate in each tertile group. ^10^ Data are presented in least square means ± standard error adjusted for age (continuous), household income, education level, current smoking, regular alcohol consumption, and physical activity.

**Table 3:** Dietary intake of macronutrients, flavonoids, and dietary total antioxidant capacity (TAC) according to tertile groups of Korean food (KF) consumption rate ^1^.

	Men			*p* for Trend ^3^	Women			*p* for Trend ^3^
	KF Consumption Rate Tertiles ^2^				KF Consumption Rate Tertiles ^2^			
Mean ± SE	T1	T2	T3		T1	T2	T3	
Energy intake (kcal/d)	2393.7 ± 21.3	2365.4 ± 24.6	2188.2 ± 23.5	<0.0001	1742.0 ± 15.1	1709.3 ± 14.1	1613.4 ± 15.8	<0.0001
% Energy from								
Carbohydrate	58.4 ± 0.3	60.5 ± 0.4	61.9 ± 0.4	<0.0001	61.4 ± 0.3	66.2 ± 0.3	69.1 ± 0.3	<0.0001
Fat	23.4 ± 0.2	19.4 ± 0.2	16.7 ± 0.2	<0.0001	23.9 ± 0.2	19.2 ± 0.2	16.2 ± 0.2	<0.0001
Protein	14.4 ± 0.1	14.6 ± 0.1	14.4 ± 0.1	0.3646	14.2 ± 0.1	14.4 ± 0.1	14.2 ± 0.1	0.0998
Total flavonoids intake (mg/1000 kcal/d)	77.42 ± 4.04	97.44 ± 3.53	100.56 ± 3.13	<0.0001	108.34 ± 4.96	136.39 ± 4.61	150.10 ± 4.96	<0.0001
Flavonol	9.95 ± 0.25	13.28 ± 0.42	16.09 ± 0.79	<0.0001	10.67 ± 0.29	14.16 ± 0.42	16.36 ± 0.64	<0.0001
Flavone	0.63 ± 0.15	0.54 ± 0.02	0.62 ± 0.02	0.8730	0.66 ± 0.09	0.65 ± 0.02	0.64 ± 0.02	0.8893
Flavanone	3.25 ± 0.29	2.86 ± 0.25	2.13 ± 0.21	0.0028	6.24 ± 0.52	5.83 ± 0.45	4.26 ± 0.30	0.0028
Flavan-3-ol	9.77 ± 2.82	9.84 ± 1.94	7.17 ± 0.64	0.4598	17.36 ± 3.85	10.87 ± 0.65	11.15 ± 0.63	0.1084
Isoflavone	6.42 ± 0.29	9.00 ± 0.35	10.43 ± 0.40	<0.0001	6.71 ± 0.30	9.24 ± 0.30	10.49 ± 0.43	<0.0001
Anthocyanidin	18.29 ± 1.05	19.05 ± 1.08	21.64 ± 1.39	0.0601	21.75 ± 1.00	29.06 ± 1.64	34.93 ± 2.39	<0.0001
Proanthocyanidin	29.12 ± 1.97	42.87 ± 1.98	42.49 ± 1.98	<0.0001	44.95 ± 1.84	66.59 ± 3.49	72.27 ± 3.02	<0.0001
Dietary TAC (mg VCE/1000 kcal/d) ^4^	137.25 ± 5.75	169.86 ± 5.43	186.41 ± 5.37	<0.0001	191.88 ± 7.62	240.19 ± 7.10	272.61 ± 8.23	<0.0001

^1^ All analyses accounted for the complex sampling design effect and considered strata, clusters, and weights. ^2^ The KF consumption rate was calculated as the proportion of KF among all food consumed by a subject in a single day and stratified into tertile groups. ^3^ *P* for trend values were obtained based on the median value for the KF consumption rate in each tertile. ^4^ VCE: vitamin C equivalents.

**Table 4:** Odds ratios (ORs) and 95% confidence intervals (CIs) of elevated C-reactive protein levels according to Korean food (KF) consumption rate tertile group and flavonoid intake or dietary total antioxidant capacity (TAC) ^1^.

	Men			Women		
	KF Consumption Rate Tertile ^2^			KF Consumption Rate Tertile ^2^		
OR (95% CI) ^3^	T1	T2	T3	T1	T2	T3
Total flavonoids intake						
<Median	1.00 (Ref)	1.35 (0.94, 1.95)	1.22 (0.84, 1.78)	1.00 (Ref)	0.91 (0.67, 1.25)	0.92 (0.64, 1.31)
≥Median	1.07 (0.72, 1.60)	1.02 (0.69, 1.50)	1.01 (0.69, 1.47)	0.92 (0.64, 1.31)	0.91 (0.65, 1.28)	0.59 (0.42, 0.83)
Flavonol intake						
<Median	1.00 (Ref)	1.10 (0.75, 1.62)	1.29 (0.90, 1.85)	1.00 (Ref)	0.77 (0.55, 1.07)	0.76 (0.53, 1.08)
≥Median	0.96 (0.66, 1.39)	1.16 (0.80, 1.67)	0.88 (0.60, 1.29)	0.83 (0.59, 1.18)	0.99 (0.72, 1.37)	0.67 (0.48, 0.94)
Flavone intake						
<Median	1.00 (Ref)	1.16 (0.79, 1.70)	1.23 (0.86, 1.76)	1.00 (Ref)	0.85 (0.59, 1.20)	0.85 (0.60, 1.21)
≥Median	0.96 (0.66, 1.39)	1.11 (0.77, 1.58)	0.95 (0.65, 1.39)	1.04 (0.72, 1.51)	1.08 (0.79, 1.48)	0.72 (0.51, 0.99)
Flavanone intake						
<Median	1.00 (Ref)	1.14 (0.78, 1.67)	1.37 (0.94, 1.99)	1.00 (Ref)	0.71 (0.51, 0.99)	0.67 (0.48, 0.96)
≥Median	1.10 (0.76, 1.59)	1.28 (0.86, 1.89)	0.92 (0.63, 1.36)	0.73 (0.53, 1.02)	0.92 (0.66, 1.29)	0.63 (0.44, 0.92)
Flavan-3-ol intake						
<Median	1.00 (Ref)	1.29 (0.89, 1.87)	1.17 (0.80, 1.71)	1.00 (Ref)	0.93 (0.67, 1.28)	0.92 (0.65, 1.29)
≥Median	0.91 (0.60, 1.38)	0.93 (0.64, 1.33)	0.88 (0.61, 1.28)	1.00 (0.71, 1.40)	0.97 (0.69, 1.36)	0.62 (0.44, 0.89)
Isoflavone intake						
<Median	1.00 (Ref)	1.39 (0.94, 2.04)	1.28 (0.89, 1.84)	1.00 (Ref)	0.94 (0.68, 1.29)	0.87 (0.62, 1.24)
≥Median	1.20 (0.80, 1.81)	1.11 (0.76, 1.63)	1.08 (0.73, 1.59)	0.99 (0.70, 1.39)	0.95 (0.70, 1.28)	0.66 (0.48, 0.92)
Anthocyanidin intake						
<Median	1.00 (Ref)	1.42 (0.97, 2.07)	1.52 (1.04, 2.22)	1.00 (Ref)	0.80 (0.58, 1.11)	0.80 (0.57, 1.12)
≥Median	1.44 (0.97, 2.13)	1.36 (0.91, 2.02)	1.10 (0.73, 1.64)	0.73 (0.52, 1.03)	0.85 (0.62, 1.17)	0.54 (0.38, 0.79)
Proanthocyanidin intake						
<Median	1.00 (Ref)	1.26 (0.88, 1.82)	1.20 (0.84, 1.72)	1.00 (Ref)	0.84 (0.61, 1.15)	0.98 (0.69, 1.39)
≥Median	0.85 (0.56, 1.30)	0.89 (0.62, 1.29)	0.80 (0.55, 1.16)	0.90 (0.64, 1.27)	0.97 (0.70, 1.36)	0.52 (0.37, 0.73)
Dietary TAC						
<Median	1.00 (Ref)	1.32 (0.91, 1.91)	1.20 (0.82, 1.75)	1.00 (Ref)	0.96 (0.68, 1.34)	0.96 (0.68, 1.36)
≥Median	1.14 (0.78, 1.68)	1.11 (0.76, 1.62)	1.10 (0.76, 1.58)	0.95 (0.67, 1.35)	0.89 (0.65, 1.24)	0.58 (0.41, 0.82)

^1^ All analyses accounted for the complex sampling design effect and considered strata, clusters, and weights. Flavonoid intake and dietary TAC were stratified based on nutrient density per 1000 kcal/d. ^2^ The KF consumption rate was calculated as the proportion of KF among all food consumed by a subject in a single day and stratified into tertile groups. ^3^ ORs and 95% CIs were adjusted for age (continuous), household income, education level, current smoking, regular alcohol consumption, and physical activity.
